# Stability indicating RP-HPLC technique for simultaneous estimation of nirmatrelvir and ritonavir in their new copackaged dosage form for COVID-19 treatment

**DOI:** 10.1038/s41598-025-85776-8

**Published:** 2025-01-17

**Authors:** Mohamed G. Yassin, Aya Roshdy, Aya A. Marie

**Affiliations:** 1 Zeta Pharma for Pharmaceutical Industries, Sadat City, Egypt; 2 Pharmaceutical Chemistry Department, Faculty of Pharmacy, Horus University, New Damietta, 34517 Egypt

**Keywords:** Nirmatrelvir (NIR), Ritonavir (RIT), RP-HPLC, Stability indicating, Copackaged tablet, Molecular medicine, Chemistry

## Abstract

RP-HPLC technique was developed and optimized for simultaneous identification and estimation of nirmatrelvir (NIR) and ritonavir (RIT) in their new copackaged tablet. Stability of nirmatrelvir (NIR) was studied after exposure to different five stress conditions; alkali, acid, heat, photo and oxidation degradation. The chromatographic separation was achieved using VDSpher PUR 100 ODS (4.6-mm x 15-mm), 3.5 μm column and mixture of 0.03 M potassium di-hydrogen phosphate buffer pH 4 and acetonitrile (45:55, v/v) as mobile phase. The column temperature was set at 40 °C, flow rate at 1mL/min and UV detection at 215 nm. The NIR and RIT retention times were 3.94 ± 0.08 min and 9.08 ± 0.1 min, respectively. Linear relationship was established in range of (1.5–105 µg/mL) for NIR and (1–70 µg/mL) for RIT with good reproducibility. The found mean percentage recoveries of nirmatrelvir (NIR) and ritonavir (RIT) were 100.03% and 99.85%, respectively. The developed method shows very good sensitivity as the LOQ and LOD were found to be 3.001 & 0.990 µg/mL, respectively for NIR and 2.765 & 0.912 µg/mL, respectively for RIT. The developed approach was validated concerning to ICH guidelines and applied successfully for the simultaneous estimation of NIR and RIT in their new copackaged dosage from. The results of assay using the proposed approach were compared statistically to the results found by applying the published one with good agreement.

**Supplementary Information:**

The online version contains supplementary material available at 10.1038/s41598-025-85776-8.

## 1. Introduction

The coronavirus (COVID-19) has widely spread all over the world from 2019 until now. The overall number of infected people globally had exceeded 519 million concerning to World Health Organization (WHO) as of May 18, 2022^1^. Even though many vaccines are commercially available, the new variants of the virus keep emerging due to the easy mutation of COVID-19, making the development of new drugs and vaccines very important^2^.

The COVID-19 is disturbing the whole world, including Egypt. It is caused by the severe acute respiratory syndrome Corona virus 2 (SARS-CoV-2)^3^.

NIR (Fig**.**S1a) is a major protease inhibitor for corona virus, NIR is an inhibitor of cytochrome P4503A (CYP3A)^4^. Numerous analytical approaches have been published for estimation of NIR alone and in combinations^5–8^.

RIT (Fig**.****S1**b**)**increases plasma concentrations of NIR via rapid and potent inhibition of the cytochrome P450 (CYP) 3A4 which is responsible for NIR metabolism^9,10^. So it’s used as a pharmacokinetic enhancer, boosts the plasma concentration of NIR and prolonging its half-life when the two drugs are administered together^11^.

Numerous analytical techniques were developed for analysis of RIT alone^12–17^ and in combination involving spectrophotometry^18–20^, HPLC^21–23^ and HPTLC^24^ methods.

Zetapaxovir tablet is a copackaged antiviral tablet containing NIR and RIT in ratio (3:1) for treatment of COVID-19. Zetapaxovir tablet is composed of 150 mg NIR per tablet and 100 mg RIT per table. It was developed by Zeta Pharma for Pharmaceutical Industries. Zetapaxovir aids to control patients with mild or moderate COVID-19. NIR is absorbed rapidly when it is administered alone, with peak plasma concentration occurring about 3 h^25^. While co-administration of NIR with RIT increases plasma concentrations of NIR and prolongs its half-life; half-life of NIR ≈ 6 h when administered with RIT^25^.

Few analytical approaches were published for the simultaneous analysis of NIR and RIT in their copackaged tablet. These techniques involved one spectrofluorimetric method^26^, LC-MS methods^27–31^, two HPLC methods^32,33^ and HPTLC method^34^. The proposed approach was able to estimate both NIR and RIT without needing to tedious methods like LC–MS.

The reported method^32^ applied for determination of both drugs in human plasma only and not applied for tablets. The proposed method shows very good sensitivity compared to the reported RP-HPLC method^33^. Reported methods^32,33^ used less green mobile phase and solvent compared to the proposed method. The proposed method used ethanol as greener solvent than methanol used in both reported methods^32,33^.

RP-HPLC is a very powerful and rapid technique, so the first goal of this research is to develop and validate RP-HPLC technique for analyzing NIR as the main drug responsible for inhibition of cytochromeP4503A (CYP3A) in presence of RIT which aids the raise of plasma concentrations of NIR^4,9^ (goal:1).

The results of assay of NIR and RIT using the proposed RP-HPLC approach were compared statistically to those found by the reported one^33^ with good agreement.

Second goal of this work is to examine the inherent stability characters of NIR according to ICH guidelines^35^ under different five degradation conditions: alkali, acid, heat, photo and oxidation degradation as the main drug responsible for the efficacy (goal: 2).

The proposed method possess an advantage over the reported ones^32,33^ in its capability for the simultaneous estimation of NIR and RIT with higher sensitivity in a wide linear ranges.

## 2. Materials and methods

### 2.1. Apparatus and software

Shimadzu Prominence-i Series LC-2030 C 3D plus system (Shimadzu, Kyoto, Japan), with Quaternary RS pump, RS auto-sampler injector and thermostated RS column compartment. VDSpher PUR 100 ODS (4.6-mm x 15-mm), 3.5 μm column. The data acquisition was carried out using Lab Solutions Software (Shimadzu, Japan). Hettich centrifuge (Tuttlingen, Germany) and Jenway 3510 pH-meter (UK). The nylon membrane filter 0.22 μm (Millipore, Ireland) was used for buffer solution filtration before use. Rocker 811 lab vacuum pump (Lingya Dist., Kaohsiung city 802, Taiwan).

### 2.2. Chromatographic conditions

Separation was achieved using VDSpher PUR 100 ODS (4.6-mm x 15-mm), 3.5 μm column as stationary phase. Acetonitrile and 0.03 M potassium di-hydrogen phosphate buffer pH 4 in ratio (55:45, v/v) as mobile phase with UV detection at 215 nm. The flow rate was 1 mL/min and column temperature 40 °C. 5 µL was injected from each solution, chromatograms were recorded and the responses for NIR and RIT peaks were measured.

### 2.3. Materials and reagents

NIR (99.50%purity) and RIT (98.60% purity) were kindly obtained from Zeta Pharma for Pharmaceutical Industries, El Menofia, Egypt. NIR solubility in ethanol was (16.5–33) mg/mL^36^. RIT solubility in ethanol was 165 mg/mL^36^. NIR and RIT solutions were stable for one month when kept at 4 °C.

Zetapaxovir, a copackaged tablets for COVID-19; composed of NIR and RIT in (3:2) ratio. Zetapaxovir, a copackaged tablets containing 150 mg NIR and 100 mg RIT, it was obtained from Zeta Pharma for Pharmaceutical Industries. Zetapaxovir aids to control patients with mild or moderate COVID-19.

HPLC grade methanol and ethanol were bought from (Fisher, UK). Orthophosphoric acid (analytical grade) was obtained from Sigma-Aldrich. Potassium di-hydrogen phosphate (Inter. Trade Co, Japan).

### 2.4. Stock and working standard solutions

Stock standard solutions (1000 µg/mL) NIR and RIT were separately prepared in two 50mL volumetric flasks by transferring accurately weighed 50 mg of NIR and RIT into two 50 mL volumetric flasks. Powders were dissolved using ethanol then solutions were completed up to the volume with ethanol.

Serial dilution of the stock solutions for the preparation of working solutions using ethanol to obtain 75 µg/mL of NIR and 50 µg/mL of RIT. All solutions were kept at 4 °C.

### 2.5. Construction of calibration curves

Different volumes of NIR & RIT previously mentioned working solutions were transferred into 10 mL volumetric flasks separately. Then dilution of each solution using mobile phase to get solutions within concentration range (1.5–105 µg/mL) of NIR and (1–70 µg/mL) of RIT. From each solution 5 µL were injected into chromatograph under the specified separation conditions. The regression equations were obtained by plotting the average peak area versus different concentrations of NIR and RIT and the.

### 2.6. Procedure for analysis of Zetapaxovir copackaged tablets

Twenty tablets of each NIR and RIT were weighed and finely powdered separately in two separate mortars. Then accurate weight of the powders equivalent to the average weight of one tablet of each were transfer into two 200-mL volumetric flasks, add 5 mL water, swirl, add 150 ml ethanol and sonicate with shaking for 20 min. The resulted solutions were cooled then completed to volume with ethanol. The solutions were centrifuged at 5000 rpm for 15 min, then 5 mL of each supernatant was transfer into two 50mL volumetric flasks. The solutions were completed with ethanol up to the volume to obtain 75 µg/mL NIR and 50 µg/mL RIT.

### 2.7. Solutions for forced degradation studies

Stability studies were caried out to confirm the specificity of the proposed technique which was assisted by comparing the chromatogram of non-stressed 75 µg/mL NIR with those of solutions which were exposed to forced degradation conditions.

Working standard solution for degradation (375 µg/mL NIR) was prepared from stock standard (1000 µg/mL) NIR solution. The unstressed standard solution was prepared by transfer of 2 mL from (375 µg/mL NIR) into 10 mL volumetric flask and completed up to the mark using ethanol.

Comparison was carried out between the response of all degradation solutions with standard solution of 75 µg/mL NIR to determine the reduction in the NIR peak area or the appearance of any new foreign peaks.

#### 2.7.1. Alkaline hydrolysis

For examination of alkaline degradation; 2 mL from (375 µg/mL) NIR solution were transferred into 10 mL volumetric flask contain 2 mL of 1 N NaOH for (2 h.) at room temperature in dark. The resulted solution was neutralized after specified time using 2 mL of 1 N HCl and completed up to the volume with ethanol to attain 75 µg/mL NIR.

#### 2.7.2. Acid degradation

For examination of acidic degradation; 2 mL from (375 µg/mL) NIR solution were transferred into 10mL volumetric flask contain 2 mL of 1 N HCl for (2 h.) in dark at room temperature. The resulted solution was neutralized after specified time using 2 mL from 1 N NaOH and completed up to the volume with ethanol to attain 75 µg/mL NIR.

#### 2.7.3. Oxidation

Oxidation degradation was exanimated by transferring 2 mL from (375 µg/ml) NIR solution into 10mL volumetric flask, this solution was exposed to 2 mL of 3% v/v H_**2**_O_**2**_ and kept in dark for 2 h at room temperature. Then solution was completed up to the volume with ethanol.

#### 2.7.4. Photo degradation

Photodegradation was exanimated by transferring 2 mL of (375 µg/mL) NIR into 10 mL volumetric flask and the solution was completed to the volume with ethanol then exposed to the sun light for 2 h. After the mentioned time the solution was injected into chromatograph.

#### 2.7.5. Heat degradation

Heat degradation was exanimated by transferring 2 mL of (375 µg/ml) NIR into 10mL volumetric flask and the solution was completed up to the volume with ethanol then exposed to 80 °C for 2 h.

## 3. Results and discussion

**Fig. 1:**
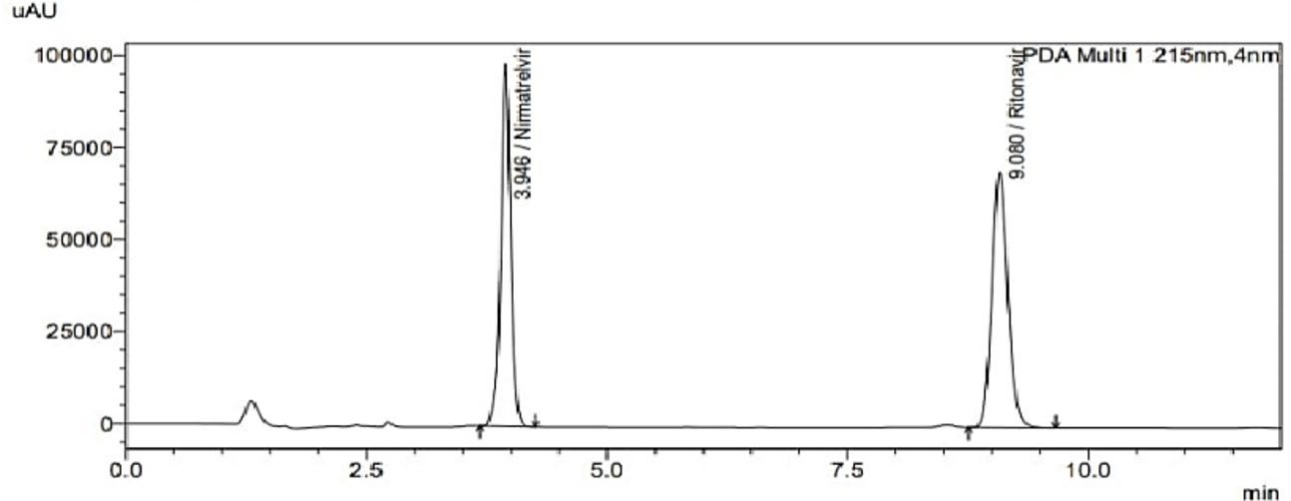
Typical chromatogram of separated drugs 75 µg/mL of NIR and 50 µg/mL RIT.

The proposed approach was successfully used to separate the binary mixture of NIR and RIT in their pure forms and copackaged tablet dosage forms within 10 min using acetonitrile and 0.03 M potassium di-hydrogen phosphate buffer pH 4 (55:45, v/v) as mobile phase. The retention times of NIR and RIT were 4.946 ± 0.08 min and 9.087 ± 0.1 min, respectively as shown in Fig. 1.

### 3.1. Method optimization

Numerous parameters were known to affect the separation, resolution and peak shape such as: buffer pH, organic modifier ratio, flow rate and temperature. These parameters were examined and gauged concerning to retention time, number of theoretical plates, resolution value and tailing factor.

#### 3.1.1. Effect of acetonitrile ratio

The ratio of acetonitrile was changed between (30 − 80%) at fixed flow rate 1mL/min, pH 4 and temperature 40 °C. The ratio 55% was carefully chosen as the best ratio for optimum resolution and separation of NIR and RIT.

#### 3.1.2. Effect of column temperature

Different temperature values were investigated at fixed flow rate 1mL/min, pH 4 and 55% acetonitrile. As the temperature increased up to 40 °C gave more better peaks shape. So, 40 °C was carefully chosen as the optimal column temperature.

#### 3.1.3. Effect of flow rate

The flow rate was studied in range (0.9–1.5) mL/min. The results showed that the flow rate less than 1mL/min gave peak broadening and too late retention times of both drugs. While using flow rate more than 1mL/min gave minimum resolution. So, 1mL/min was carefully chosen as the optimal flow rate.

#### 3.1.4. Effect of pH of buffer

Different pH values were tested between (3–6) at fixed acetonitrile ratio 55%, temperature was 40 °C and flow rate 1mL/min. The pH 4 was carefully selected for higher resolution and better separation.

TableS1 shows the system suitability parameters for analysis of NIR and RIT at optimum chromatographic conditions.

### 3.2. Method validation

The developed technique was validated according to ICH guidelines^37^.

#### 3.2.1. Linearity and range

NIR and RIT linearity ranges were (1.5–105 µg/mL) and (1–70 µg/mL), respectively. Table 1 shows the regression parameters for NIR and RIT which indicate good linearity for NIR and RIT where r more than 0.999.

#### 3.2.2. Limits of detection and quantitation

LOD and LOQ for NIR and RIT in their pure and copackaged tablet were calculated according to the ICH guideline^37^ by the following equations and the results are presented in Table 1.

LOD = 3.3×ơ/S (1).

LOQ = 10×ơ/S (2).

where S is the slope and ơ is the standard deviation of y-intercept of regression lines.

Table 1 showed very low values of LOD and LOQ which indicates that the proposed approach is highly sensitive for estimation of NIR and RIT.

**Table 1:** Regression parameters for estimation of NIR and RIT using the proposed technique.

Drug	NIR	RIT
Concentration range (µg/mL)	1.5–105	1–70
r	0.9999	0.9998
a	−3825.707	−8692.963
b	9214.947	15988.118
S_a_	2765.123	4420.401
S_b_	49.288	118.189
S_(y/x)_	5634.766	9007.890
LOD (µg/mL)	0.990	0.912
LOQ (µg/mL)	3.001	2.765

a, intercept; b, slope; r, correlation coefficient.

S_a_, standard deviation of intercept; S_b_, standard deviation of slope;.

S_y/x_, residual standard deviation.

LOD, limit of detection; LOQ, limit of quantitation.

#### 3.2.3. Accuracy “Trueness”

Trueness of the proposed approach was assisted via calculation of mean % recoveries of NIR and RIT of triplicate determination of at three different concentrations level within the linearity ranges. For NIR (60, 75 and 90 µg/mL) and for RIT (40, 50 and 60 µg/mL). Table S2 shows the calculated mean % recovery values for NIR and RIT which were found to be within compendial tolerance (98–102%).

#### 3.2.4. Precision

Intraday precision was evaluated by calculating (S.D) and (% RSD) for triplicate injections of different three concentrations of NIR and RIT on the same day and on three successive days for inter-day precision. The % RSD was less than 2 for both drugs as shown in Table S3 indicating good inter and intraday precisions of the developed approach.

#### 3.2.5. Selectivity

The analytical method is selective according to ICH guidelines^37^, when it is unaffected by the different excipients present in dosage forms.

**Fig. 2:**
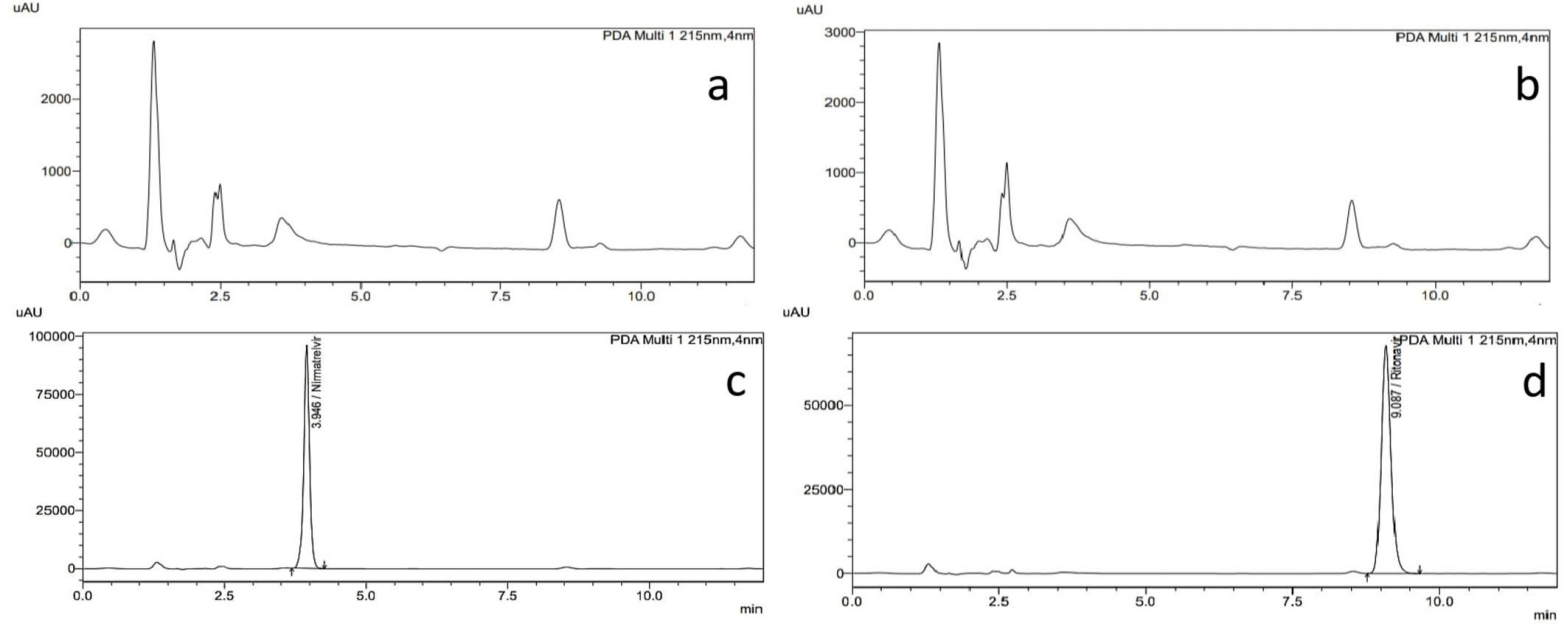
Chromatograms of **a** placebo of RIT tablet, **b** placebo of NIR tablet, **c** 75 µg/mL NIR tablets, **d** 50 µg/mL RIT tablets.

The selectivity evaluated by comparing the chromatograms of the placebo of both drugs separately contain all excipients with the chromatograms of the standard pure drugs at the optimum chromatographic conditions**. **Figure 2 shows that there is no interference from the excipients with the candidate drugs.

#### 3.2.6. Robustness

The developed approach was robust since it was persisted unaffected by the small deliberate changes of different factors. Table S4 shows that the resulted values of S.D. and % RSD of %recovery were less than 2 which confirm the robustness of proposed approach for estimation of both drugs.

### 3.3. Results of forced degradation studies

**Fig. 3:**
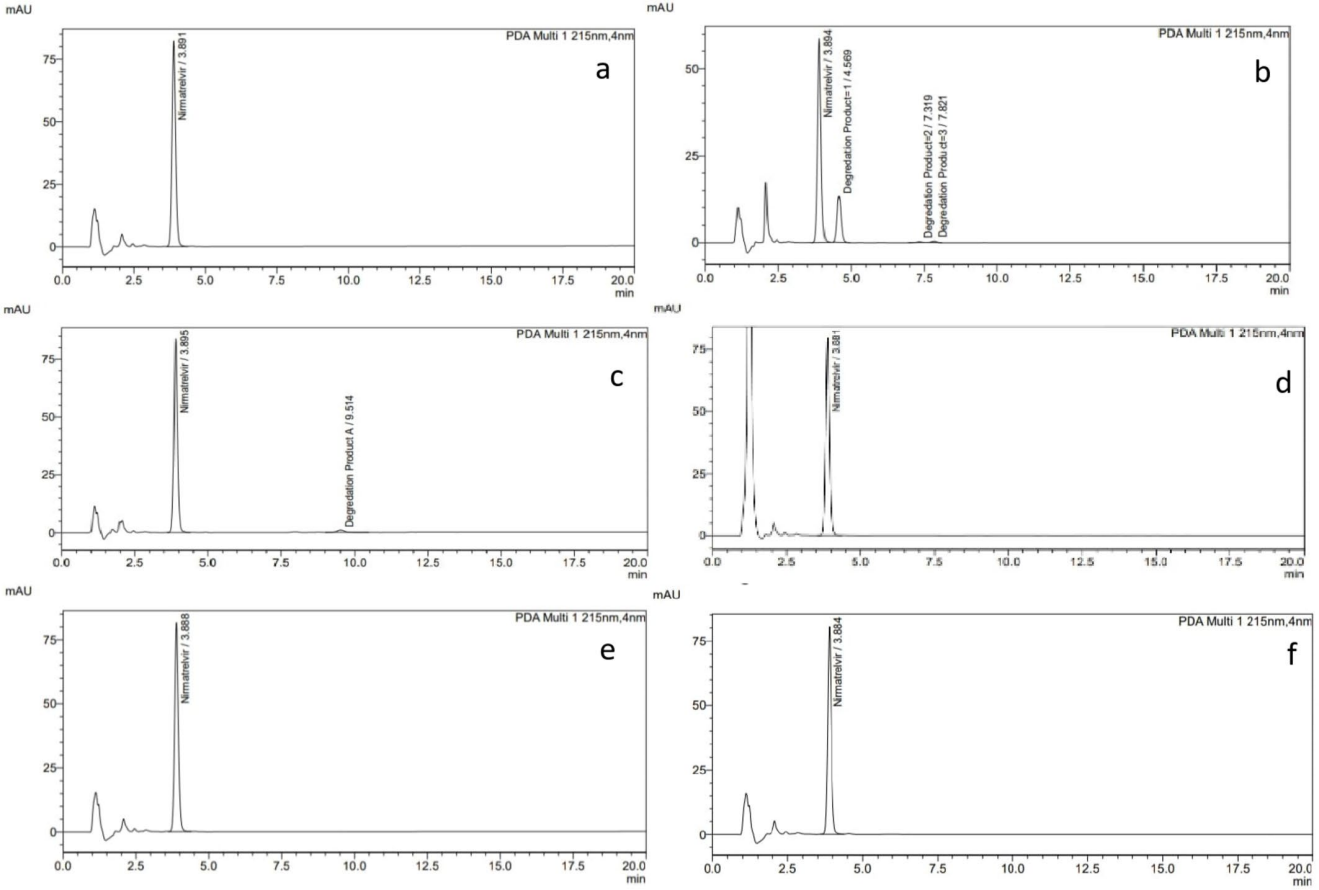
**a** chromatogram of a non-stressed standard solution of 75 µg/mL NIR, **b **chromatogram of degradation under alkaline hydrolysis using 1 N NaOH, **c** chromatogram of degradation under acidic hydrolysis using 1 N HCL, **d** chromatogram of 75 µg/mL NIR under oxidative degradation condition, **e **chromatogram of photo degradation of 75 µg/mL NIR for 2 h., **f **chromatogram of heat degradation after being exposed to heat at 80 °C for 2 h.

The specificity of the proposed technique is established by investigating the chromatogram of a non-stressed standard solution of NIR (75 µg/mL) as shown in Fig. 3(a) with those solutions which were exposed to forced degradation conditions.

Different five degradation conditions were used for examination of the inherent stability characteristics of the NIR as per ICH guidelines^35^. These conditions were; alkaline, acidic, heat, oxidation and photo degradation.

#### 3.3.1. Alkaline hydrolysis

NIR was very sensitive to alkaline degradation as it gave the maximum %degradation using 1 N NaOH. The %recovery of NIR was decreased about 31.263% and three new foreign peaks were detected as shown in Table 2; Fig. 3(b).

#### 3.3.2. Acid hydrolysis

NIR exhibited very slight acidic degradation using 1 N HCL, the %recovery of NIR was decreased only about 2.7% compared to the unstressed solution with appearance of one degradation product peak as shown in Fig. 3(c) and Table 2. This result revealed that the NIR is just slightly sensitive to the acidic degradation using 1 N HCl.

#### 3.3.3. Oxidative degradation

NIR was more stable under oxidative degradation condition compared to alkaline degradation, but still it was slightly sensitive to oxidative degradation as the degradation present was found to be about 3.4% as presented in Fig. 3(d) and Table 2.

#### 3.3.4. Photo degradation

The NIR was exhibited good stability under photo degradation conditions, this was indicated by the peak area of NIR which doesn’t decrease after being exposed to the light for 2 h and no foreign peaks appeared as a result of the this forced degradation as shown in Fig. 3(e) and Table 2.

#### 3.3.5. Heat hydrolysis

NIR showed good stability under the heat degradation condition as the peak area of NIR doesn’t decrease after being exposed to the heat at 80 °C for 2 h and no foreign peaks appeared as presented in Fig. 3(f) and Table 2.

**Table 2:** Results of forced degradation study for NIR.

Degradation conditions	Unstressed	Photolytic (Light, 2 h.)	Alkaline (1 *N* NaOH, 2 h.)	Oxidation (3%H_2_O_2_, 2 h.)	Heat (80 °C, 2 h.)	Acidic 1 *N* HCl, 2 h.
Peak Area	721,982	719,122	496,272	697,499	706,915	702,556
% Found	100.00%	99.604	68.737	96.609	97.913	97.309
% Degradation	--	0.396	31.263	3.391	2.087	2.691
New peaks	--	--	3 Peaks	--	--	1 Peak

### 3.4. Assay of Zetapaxovir copackaged tablets

**Fig. 4:**
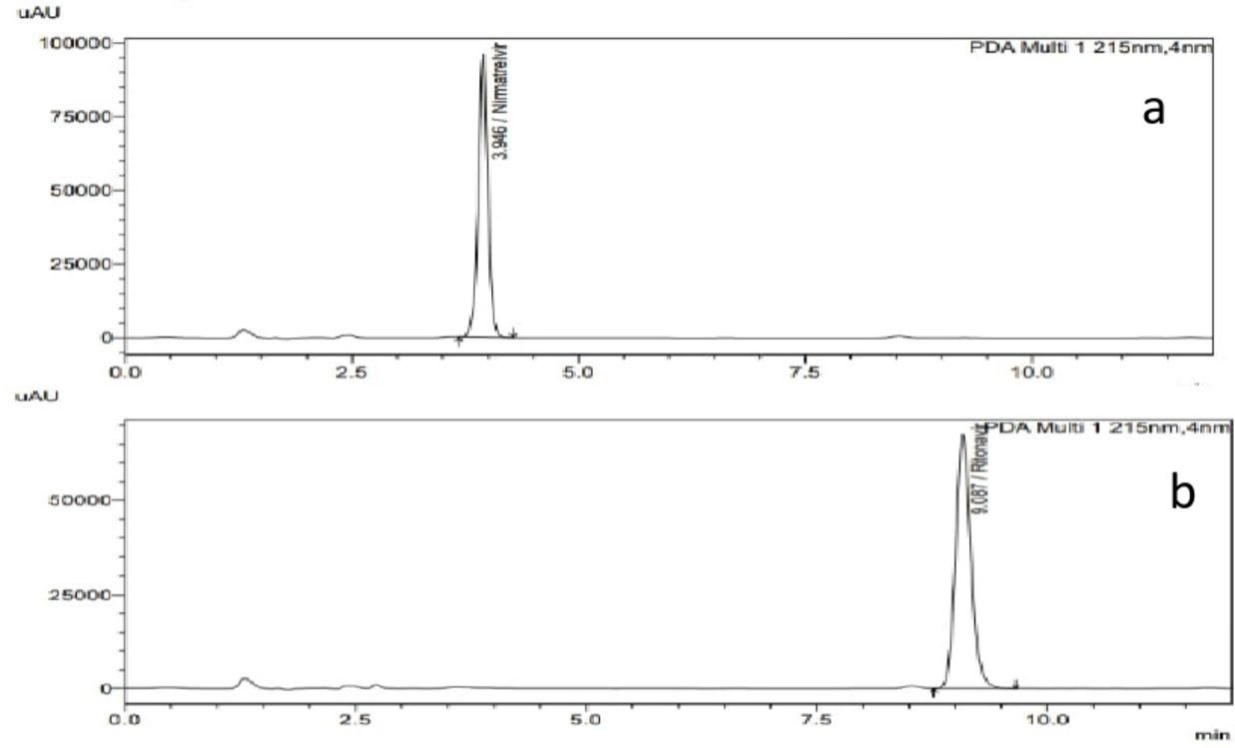
Separated chromatograms of **a** 75 µg/mL NIR tablet and **b** 50 µg/mL RIT tablet in Zetapaxovir copackaged tablets.

The proposed method was applied for the simultaneous analysis of NIR and RIT in Zetapaxovir copackaged tablets that is available in Egyptian markets. Reasonable results were found for NIR and RIT with good agreement with labeled claim as presented in Fig. 4 & Table 3.

A comparison between the proposed and reported approaches was based on using T-test and F-test for accuracy and precision at 95% confidence level, respectively as represented in Table 3. The calculated values did not exceed the tabulated ones, indicates that there was no significant difference between the proposed and reported^33^ approaches.

A comparison between the proposed and reported approaches^33^ shows that the proposed technique has wider linearity range and more sensitive than reported HPLC technique.

**Table 3:** Results of assay of Zetapaxovir tablets using the proposed technique and reported one^33^.

	Proposed method		Reported Method^33^	
Drugs	NIR	RIT	NIR	RIT
Mean (Ẋ)	101.035	100.821	100.115	100.759
S.D.	0.831	0.631	0.984	0.854
%RSD	0.822	0.626	0.982	0.848
T_cal_	1.750	0.144	T _tab_	2.228
F_cal_	0.714	0.545	F _tab_	5.000

## Summary

The proposed RP-HPLC technique was developed and validated for simultaneous estimation of nirmatrelvir (NIR) and ritonavir (RIT) in their pure and new copackaged tablet forms. The chromatographic separation was achieved using VDSpher PUR 100 ODS (4.6-mm x 15-mm), 3.5 μm column. Mixture of 0.03 M potassium di-hydrogen phosphate buffer pH 4 and acetonitrile (45:55, v/v) as mobile phase. The UV detection at 215 nm. The NIR and RIT retention times were 3.94 ± 0.08 min and 9.08 ± 0.1 min, respectively. Stability of nirmatrelvir (NIR) was studied after exposure to different five stress conditions. The developed approach was validated concerning to ICH guidelines with good linear relationship was established in range of (1.5–105 µg/mL) for NIR and (1–70 µg/mL) for RIT. The found mean percentage recoveries of nirmatrelvir (NIR) and ritonavir (RIT) were 100.03% and 99.85%, respectively. The developed method shows very good sensitivity as the LOQ and LOD were found to be 3.001 & 0.990 µg/mL, respectively for NIR and 2.765 & 0.912 µg/mL, respectively for RIT. The method was applied successfully for the simultaneous estimation of NIR and RIT in their new copackaged dosage from and the results of assay were statistically compared to the results found by applying the published one with good agreement.

## Conclusion

This work presents a fast and sensitive approach for the estimation of NIR and RIT in their pure forms and in their copackaged Zetapaxovir tablets. Stability studies of NIR were caried out using five different degradation conditions to confirm the specificity of the proposed approach. The technique was suitable for analysis of NIR and RIT in pure and pharmaceutical preparations.

## Electronic supplementary material

Below is the link to the electronic supplementary material.

Supplementary Material 1

## Data Availability

All data generated or analysed during this study are included in this published article and its supplementary information files.
