# Supplementary material for: Stability indicating RP-HPLC technique for simultaneous estimation of nirmatrelvir and ritonavir in their new copackaged dosage form for COVID-19 treatment
Source: Sci Rep. 2025 Jan 17;15:2281. doi: 10.1038/s41598-025-85776-8 (PMC11748733; doi:10.1038/s41598-025-85776-8)
Supplement: Supplementary file 1 — Supplementary Material 1 [file 41598_2025_85776_MOESM1_ESM.docx]

**Supplementary Files**

Mohamed G. Yassin ^a^, Aya Roshdy ^b^, Aya A. Marie ^b^*

^a^ Zeta Pharma for Pharmaceutical Industries, Sadat City, Egypt.

^b^ Pharmaceutical Chemistry Department, Faculty of Pharmacy, Horus University, New Damietta, 34517, Egypt.

1. Mohamed G. Yassin (given name, middle, family name)

Methodology section head, Zeta Pharma for Pharmaceutical Industries, Sadat City, Egypt*.*

*Email:* [*mohamed.goda@zeta-pharma.com*](mailto:mohamed.goda@zeta-pharma.com)

*Tel. +201090941956*

1. Aya Roshdy (given name, middle, family name)

*Lecturer,* *Pharmaceutical Chemistry Department, Faculty of Pharmacy, Horus University, New Damietta, 34517, Egypt*

*Email:* [*afouda@horus.edu.eg*](mailto:afouda@horus.edu.eg)

*Tel. +201092291224*

1. Aya A. Marie***(the corresponding author)** (given name, middle, family name)

*Lecturer,* *Pharmaceutical Chemistry Department, Faculty of Pharmacy, Horus University, New Damietta, 34517, Egypt*

*Email:* [*amarie@horus.edu.eg*](mailto:amarie@horus.edu.eg)

*Tel. +201111135873*

**Supplementary figure**

**Fig. S1**: Chemical structures of NIR (a) and RIT (b)

**Supplementary tables**

**Table S1**: System suitability parameters for analysis of NIR and RIT at optimum chromatographic conditions.

**Table S2:** Results of accuracy evaluation for analysis of NIR and RIT.

**Table S3**: Results of precision for estimation of NIR and RIT.

**Table S4**: Results of robustness for estimation of NIR and RIT using the developed technique.

**Table S1**. System suitability parameters for analysis of NIR and RIT at optimum chromatographic conditions.

| Parameters | Drugs | Results | Reference values |
| --- | --- | --- | --- |
| Retention time t_R_ (min.) | NIR | 3.946 | -- |
|  | RIT | 9.087 | -- |
| Tailing factor | NIR | 0.976 | 0.8-1.2 |
|  | RIT | 1.144 |  |
| Number of theoretical plates | NIR | 6194 | more is better |
|  | RIT | 13305 |  |
| Resolution (R_s_) | NIR-RIT | 19.876 | ˃2 |

**Table S2:** Results of accuracy evaluation for analysis of NIR and RIT.

| Taken Conc. (µg/ mL) | | | NIR | | RIT | |  |
| --- | --- | --- | --- | --- | --- | --- | --- |
| NIR | RIT | | Obtained Conc. (µg/ mL) | Recovery % | Obtained Conc. (µg/ mL) | Recovery % |  |
|  |  |  |  |  |  |  |  |
| 60 | | 40 | 59.048 | 98.414 | 39.304 | 98.260 |  |
| 75 | | 50 | 76.331 | 101.775 | 50.802 | 101.604 |  |
| 90 | | 60 | 89.929 | 99.921 | 59.813 | 99.688 |  |
| Mean % | | | 100.037 | | 99.851 | |  |
| S.D. | | | 1.684 | | 1.678 | |  |
| RSD % | | | 1.683 | | 1.680 | |  |

**Table S3**: Results of precision for estimation of NIR and RIT.

|  |  | Intra-day precision | | | Inter-day precision | | |
| --- | --- | --- | --- | --- | --- | --- | --- |
|  | Concentration taken (µg/mL) | Mean conc. found | S.D. | %RSD | Mean conc. found | S.D. | %RSD |
| NIR | 60 | 59.068 | 0.383 | 0.648 | 58.998 | 0.061 | 0.104 |
|  | 75 | 75.521 | 0.512 | 0.678 | 76.187 | 0.125 | 0.164 |
|  | 90 | 89.912 | 0.091 | 0.102 | 89.828 | 0.094 | 0.105 |
| RIT | 40 | 39.531 | 0.319 | 0.808 | 39.263 | 0.043 | 0.109 |
|  | 50 | 50.403 | 0.237 | 0.471 | 50.706 | 0.083 | 0.164 |
|  | 60 | 59.913 | 0.205 | 0.342 | 59.745 | 0.059 | 0.099 |

S.D: standard deviation, % RSD: percent relative standard deviation

**Table S4**: Results of robustness for estimation of NIR and RIT using the developed technique.

| Parameters | Conditions | Mean % recovery | | S.D. | | %RSD | |
| --- | --- | --- | --- | --- | --- | --- | --- |
|  |  | NIR | RIT | NIR | RIT | NIR | RIT |
| Flow rate | 0.9 mL/min | 100.531 | 101.432 | 0.882 | 0.113 | 0.877 | 0.111 |
|  | *1 mL/min |  |  |  |  |  |  |
|  | 1 mL/min |  |  |  |  |  |  |
| Temp | 38 °C | 101.599 | 101.129 | 0.417 | 0.588 | 0.411 | 0.582 |
|  | *40 °C |  |  |  |  |  |  |
|  | 42 °C |  |  |  |  |  |  |
